# Amniotic Membrane Restores Chronic Wound Features to Normal in a Keratinocyte TGF-β-Chronified Cell Model

**DOI:** 10.3390/ijms24076210

**Published:** 2023-03-25

**Authors:** Sergio Liarte, Ángel Bernabé-García, Mónica Rodríguez-Valiente, José M. Moraleda, Gregorio Castellanos, Francisco J. Nicolás

**Affiliations:** 1 Laboratorio de Regeneración, Oncología Molecular y TGF-β, IMIB-Arrixaca, El Palmar, 30120 Murcia, Spain; 2 Advanced Therapies in Regenerative Medicine Based on Molecular and Cellular Biology, Universidad Católica de Murcia (UCAM), Campus de los Jerónimos, Guadalupe, 30107 Murcia, Spain; 3 Unidad de Terapia Celular, Hospital Clínico Universitario Virgen de la Arrixaca, El Palmar, 30120 Murcia, Spain; 4 Unidad de Heridas Crónicas y Úlcera de Pie Diabético, Hospital Clínico Universitario Virgen de la Arrixaca, El Palmar, 30120 Murcia, Spain

**Keywords:** chronic wounds, cell models, amniotic membrane, wound healing, keratinocytes, TGF-β, cell proliferation, cell migration

## Abstract

Unsuccessful wound closure in chronic wounds can be linked to altered keratinocyte activation and their inability to re-epithelize. Suggested mechanisms driving this impairment involve unbalanced cytokine signaling. However, the molecular events leading to these aberrant responses are poorly understood. Among cytokines affecting keratinocyte responses, Transforming Growth Factor-β (TFG-β) is thought to have a great impact. In this study, we have used a previously characterized skin epidermal in vitro model, HaCaT cells continuously exposed to TGF-β1, to study the wound recovery capabilities of chronified/senescent keratinocytes. In this setting, chronified keratinocytes show decreased migration and reduced activation in response to injury. Amniotic membrane (AM) has been used successfully to manage unresponsive complicated wounds. In our in vitro setting, AM treatment of chronified keratinocytes re-enabled migration in the early stages of wound healing, also promoting proliferation at later stages. Interestingly, when checking the gene expression of markers known to be altered in TGF-β chronified cells and involved in cell cycle regulation, early migratory responses, senescence, and chronic inflammation, we discovered that AM treatment seemed to reset back to keratinocyte status. The analysis of the evolution of both the levels of keratinocyte activation marker cytokeratin 17 and the spatial-temporal expression pattern of the proliferation marker Ki-67 in human in vivo biopsy samples suggests that responses to AM recorded in TGF-β chronified HaCaT cells would be homologous to those of resident keratinocytes in chronic wounds. All these results provide further evidence that sustained TGF-β might play a key role in wound chronification and postulate the validity of our TGF-β chronified HaCaT in vitro model for the study of chronic wound physiology.

## 1. Introduction

The epidermis is the outermost layer of the skin. It consists of an ever-developing layer of shedding keratinocytes, whose main function is conforming a functional barrier against environmental insult. When the skin is traumatically wounded, the integrity of the epithelial barrier is lost invariably, even though the damage may affect deeper tissue layers [1]. Upon integrity degradation, keratinocytes experience sequential proliferation and migration in order to restore epidermal continuity. These responses are known to be coordinated by a variety of growth factors and cytokines (i.e., TGF-β, EGF, VEGF, PDEGF) produced by skin cells as well as inflammatory-related cells. The presence of these factors evolves in space and time, thus conditioning the responses of all cell types participating in tissue restoration throughout the sequence of overlapping healing phases: (i) inflammation, (ii) proliferation, and (iii) remodeling [2]. Complicated skin injuries that stagnate or fail to progress through these phases in an orderly manner are referred to as chronic wounds, a common complicated state in pressure ulcers, venous ulcers, diabetic ulcers, and massive traumatic wounds [3].

Among factors influencing wound healing, Transforming Growth Factor-β (TGF-β) is considered to promote the broadest spectrum of effects [4]. In that sense, accumulated evidence supports the notion that cells residing in chronic wounds can develop aberrant responses to growth factors and cytokines, including abnormal reactions to TGF-β1 [3,5,6,7]. The TGF-β family encompasses three members (β1, β2, and β3), highly conserved isoforms among vertebrates that share a strong identity and very similar three-dimensional structures. It is worth noting that all three isoforms interact with the same universally expressed transmembrane receptors, TβRI and TβRII, showing similar affinity, and thus, being space-time availability patterns, along with cell type and state, responsible for the diversity of responses [8]. In mammals, responses to TGF-β seem to be crucial for skin homeostasis, thereby playing a pivotal role in regulating the activities of every cell type involved in wound healing and repair [9]. As a reference, TGF-β1 released by platelets in acute wounds promotes the production and secretion of extracellular matrix (ECM) components, as well as fibroblast proliferation and migration. On the contrary, for keratinocytes, TGF-β1 signaling opposes the mitogenic activity of growth factors such as EGF [7].

Aberrant TGF-β signaling has been related to cutaneous conditions such as psoriasis [10] and fibrosis [11]. More importantly, there is mixed evidence on how TGF-β dynamics evolve in human skin wounds. In acute lesions, TGF-β levels are thought to sharply increase during clotting and then decrease over time throughout the healing process [6,12]. However, the available evidence obtained using human samples indicates that elevated TGF-β levels would sustain over time in the epidermis of chronic wounds, in contrast to reduced levels usually found at the dermis compartment [13,14,15]. This pattern of elevated TGF-β levels, which apparently is a hallmark of chronic ulcer epidermis, confronts the previous notion that low TGF-β levels are predominant in the whole chronic wound [8] and also suggests that excessive and prolonged TGF-β expression at the wound site would be detrimental for healing. In this sense, observations made on transgenic mice indicate that keratinocytes overexpressing TGF-β1 develop histological characteristics similar to those found in the epidermis of chronic wounds [15].

Research strategies targeting TGF-β signaling to improve human skin regeneration have historically relied on increasing TGF-β presence. Notwithstanding, the scarce existing evidence of human keratinocyte responses in vitro fails to show the positive effects of increased TGF-β levels [16,17]. In this line, recent studies in our lab allowed us to describe the morphologic and physiologic changes experienced in vitro by HaCaT keratinocytes cultured on sustained TGF-β stimulation [18]. These changes were characterized mainly by partial epithelium-to-mesenchyme transition (EMT) features and a weakened growth arrest response in the long term, along with the detection of elevated senescence and/or inflammation markers [18]. Moreover, TGF-β has been tried in a limited manner in skin pathological conditions in humans, including chronic wounds, but has failed to meet positive expectations [4,12]. However, the evidence gathered in our lab suggests that treatments capable of adjusting cell responses to TGF-β in space and time can contribute to keratinocyte activation and ultimately to resuming the healing process of chronic wounds, as is the case of treatments applying amniotic membrane (AM) [19,20]. In vitro AM treatment on regular HaCaT cells has been shown to rapidly promote focal adhesion (FA), remodeling, and cell migration [21], also modifying the genetic program induced by TGF-β in the long term [22]. Interestingly, these effects of AM might be linked to TGF-β signaling, resulting in a fine modulation of responses in time and space in relation to the wound structure [23]. It is worth highlighting that its compassionate use in chronic wounds has shown how AM treatment can promote keratinocyte proliferation and migration in vivo [22], which has proven to be extremely helpful for the assessment of massive posttraumatic wounds [20] and diabetic foot ulcers [24].

At the clinic, the clearest sign of skin wound chronification is the inability of the epidermis to complete epithelialization within a predictable time frame [25,26]. Proper keratinocyte activation responses, leading to effective proliferation and migration, are necessary for complete re-epithelization and wound closure [27]. In this sense, there is still a lack of work dealing with the potential negative effects of sustained exposure to TGF-β in chronic wounds, although it is known to profoundly condition keratinocyte physiology. In this study, we used a validated cellular model based on keratinocytes derived from human skin to address that issue. These spontaneously immortalized epithelial HaCaT cells were subjected to sustained stimulation with TGF-β1 [18] to study how this condition may impair their migration and proliferation responses upon wound challenge. Additionally, using the same model, we report on the superior ability of AM treatment to promote the restoration of proper keratinocyte responses in vitro, further providing evidence of similar molecular responses occurring in vivo in human chronic wounds treated with AM.

## 2. Results

### 2.1. HaCaT Cells under Long-Term TGF-β Treatment Show Reduced Migration Capability

In our lab, we have previously shown that, while HaCaT cells deprived of serum (SS-HaCaT) show no important changes regarding their morphology and cellular markers, HaCaT cells treated with TGF-β for a long term in the absence of serum (SSTC-HaCaT) develop several features similar to those shown by keratinocytes in chronic wounds [18]. These features, among others, include evolving from cobblestone-shaped cell islands to a clearly spread-out configuration, also growing in size [18].

**Figure 1:**
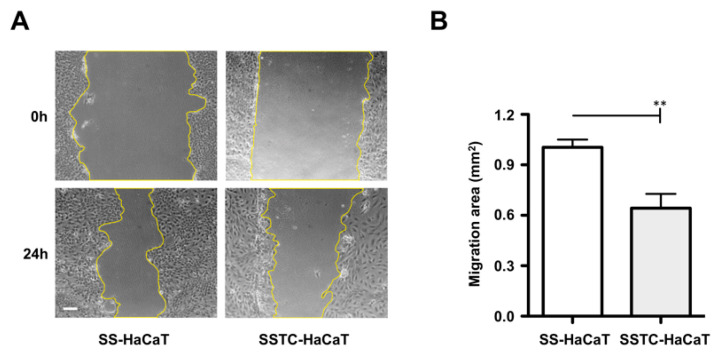
HaCaT cells persistently exposed to TGF-β show decreased migratory capacity. (**A**) Pictures representative of the migratory performance of SS-HaCaT and SSTC-HaCaT monolayers when challenged with epidermal continuity disruption. (**B**) Quantification of the migratory performance of SS-HaCaT and SSTC-HaCaT in regard to the initial gap area. Replicates from three independent experiments were analyzed. Shown data represent mean ± SEM. Asterisks denote statistically significant differences (** *p* < 0.005). The scale bar equals 100 µm.

In order to gain insight into how TGF-β chronification may affect the ability of keratinocytes to contribute to wound healing, we first performed a series of in vitro wound-healing scratch assays. Compact epithelial SS-HaCaT and SSTC-HaCaT cultures were wounded and allowed to migrate for 24 h. Upon wounding, SSTC-HaCaT cells at the wound edge quickly widened and spread, in contrast to SS-HaCaT, which remained quite the same (Figure 1A). During the assays, this change of SSTC-HaCaT cells extended beyond the wound edge, with cells exhibiting clear differences in size compared to the initial SSTC-HaCaT or SS-HaCaT cells (Figure 1A). Surprisingly, despite the increased size and greater cellular extension, SSTC-HaCaT cells exhibited a reduced ability to spontaneously migrate compared to SS-HaCaT cells (Figure 1A,B). Together, these observations indicate that the process of chronification under prolonged exposure to TGF-β did not only affect the morphology of keratinocytes, but more importantly, it also hampered their ability to migrate upon a challenge to the epithelial continuity.

### 2.2. SSTC-HaCaT Cells Show Impaired FA Dynamics When Challenged to Migrate in Response to Epithelial Wounding

**Figure 2:**
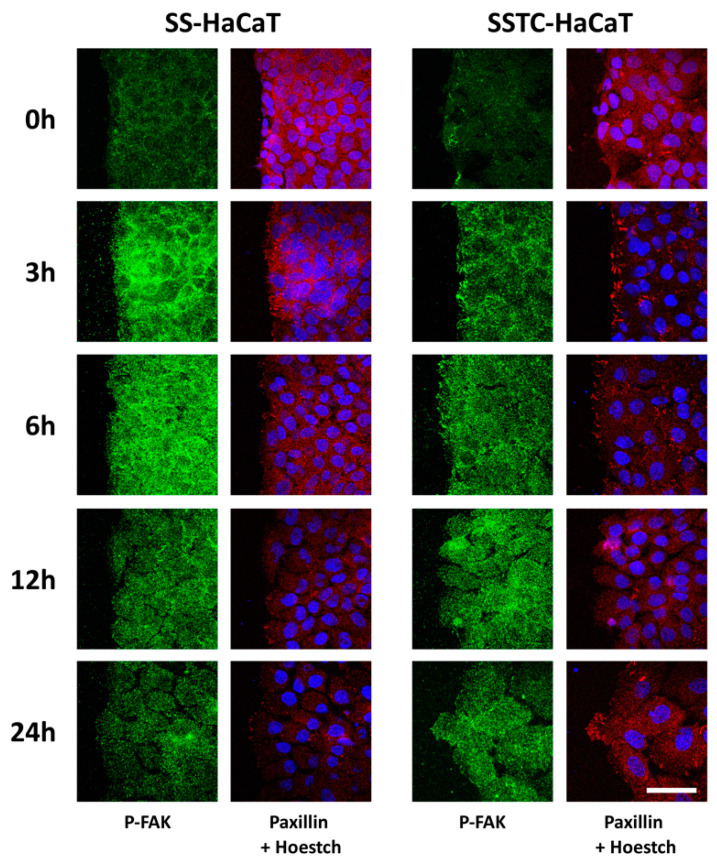
HaCaT cells persistently exposed to TGF-β showed altered FA dynamics. Distinct spatial and temporal patterns for the structural protein paxillin (red) and the activated modulator factor FAK (green) are shown in both SS-HaCaT and SSTC-HaCaT cells. Nuclei are colored in blue. Representative images of at least three independent experiments are shown. The scale bar equals 50 µm.

To understand how migration machinery responded to the wounding challenge in SSTC-HaCaT or SS-HaCaT cells, focal adhesion kinase (FAK) and FA component paxillin dynamics were examined over time. Paxillin integrates FA, and it is responsible for coordinating cell migration [28]. FA decreasing size and an increasing number have been related to positive migration [29]. The phosphorylation of FAK at residue Tyr-925 is critical for modulating FA assembly and disassembly, and it has also been related to positive cell migration [30,31,32]. Upon wounding, activation of FAK, depending on phosphorylation, and distribution of paxillin, showed unclear divergences between SSTC-HaCaT or SS-HaCaT cells, characterized by resembling FAK activities and by high availability of disorganized paxillin. However, 3 h after wounding, SSTC-HaCaT cells showed far less FAK activity than SS-HaCaT cells (Figure 2). In that sense, although FAK activity in SS-HaCaT cells seemingly peaks at 3 h to decrease over time, in the case of SSTC-HaCaT, FAK activity steadily increases to peak at about 12 h and slightly fades after that (Figure 2). In parallel, we detected the evident presence of stronger FA structures composed of paxillin at 3 h post wounding in SSTC-HaCaT cells than in SS-HaCaT cells. In fact, stronger paxillin focal points were found to be a distinct trait of SSTC-HaCaT cells compared to SS-HaCaT cells at all studied times, while paxillin availability increased over time (Figure 2). These data suggest that the lack of mobility at the migrating front, which is characteristic of SSTC-HaCaT cells, might be a consequence of altered FAK activation resulting in a suboptimal FA turn-over required for effective cell migration.

### 2.3. Amniotic Membrane Treatment Re-Enables Migration Capacities to TGF-β-Chronified HaCaT Keratinocytes

Our lab has an extensive track record on the evaluation of how epidermal growth factor (EGF), fetal bovine serum (FBS), or amniotic membrane (AM) treatments affect the capability of keratinocytes to contribute to the wound healing process [18,19,22,33]. We performed in vitro wound-healing scratch migration assays on both SS-HaCaT and SSTC-HaCaT, now introducing different treatments in the form of TGF-β, FBS and/or AM, analyzing their performance at the 24 h mark. In a similar fashion to regular HaCaT cells, SS-HaCaT cells improved their migration performance when exposed to AM alone.

**Figure 3:**
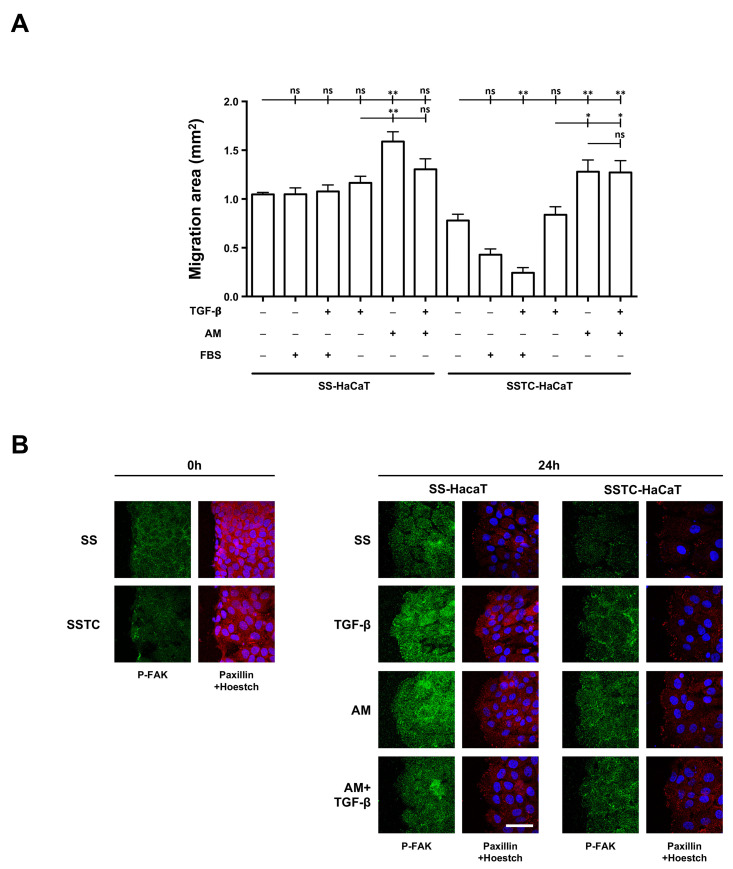
Exposure to amniotic membrane empowers migration capabilities and FA dynamics in keratinocytes persistently exposed to TGF-β. (**A**) Quantification of the migratory performance of SS-HaCaT and SSTC-HaCaT with regard to the initial gap area and the presence of adjuvant treatments. Replicates from three independent experiments were analyzed. Shown data represent the mean ± SEM. Asterisks denote statistically significant differences (* *p* < 0.05, ** *p* < 0.005). (**B**) Spatial and temporal patterns for the structural protein paxillin (red) and the activated modulator factor phosphorylated-FAK (green) in relation to SS-HaCaT and SSTC-HaCaT cell conditions and the exposure to adjuvant treatments. Nuclei are colored blue. Representative images of at least three independent experiments are shown. The scale bar equals 50 µm; ns, non-significant.

AM-TGF-β dual treatment resulted in slightly increased migration, although not enough to reach significance (Figure 3A). Contrary to previous knowledge, we found that neither TGF-β, FBS, nor respective dual treatments resulted in appreciable migration gains for SS-HaCaT cells (Figure 3A). As previously shown in Figure 1, SSTC-HaCaT cell migration was relatively slow when compared to SS-HaCaT cells. However, when AM was added to wounded cultures, the cells recovered a slightly higher migratory potential than that of untreated SS-HaCaT cells (Figure 3A). Similar to SS-HaCaTs, TGF-β did not offer any improvements for SSTC-HaCaT cells. Moreover, both FBS and FBS-TGF-β dual treatments resulted in evident reductions in cell migration. These results add to our knowledge of the capabilities of AM treatments to boost the migration of keratinocytes, in this case being able to reinstitute the migratory response of TGF-β chronified cells.

We verified whether the improvements in the migration of chronic wound cells by AM involved changes in the behavior of FAK and the dynamics of FAs. Using endpoint pictures obtained from migrating edges of SS-HaCaT cultures, we found that FAK activity, naturally induced by the wound, further increased in the presence of AM or TGF-β and only slightly in the case of simultaneous treatment. A similar trend was also found for paxillin availability in SS-HaCaT cultures (Figure 3B). On the contrary, for SSTC-HaCaT cultures, wounding did not induce or increase FAK activation. Treatment with TGF-β alone did not provide appreciable changes for any of the markers studied. However, in the case of SSTC-HaCaT cultures exposed to AM, both with or without TGF-β, there was increased FAK activity at 24 h, and paxillin availability and focal points showed lesser intensity and higher levels (Figure 3B). These results point to the ability of AM treatments to re-enable proper functional responses and turn-over of FA in SSTC-HaCaT cells, which is fully coherent with an AM restoration of the migration ability of SSTC-HaCaT cells.

### 2.4. Reinstituted Wound Recovery Capacity Is Independent of Changes in Cell Morphology and Proliferation in the Early Stages

**Figure 4:**
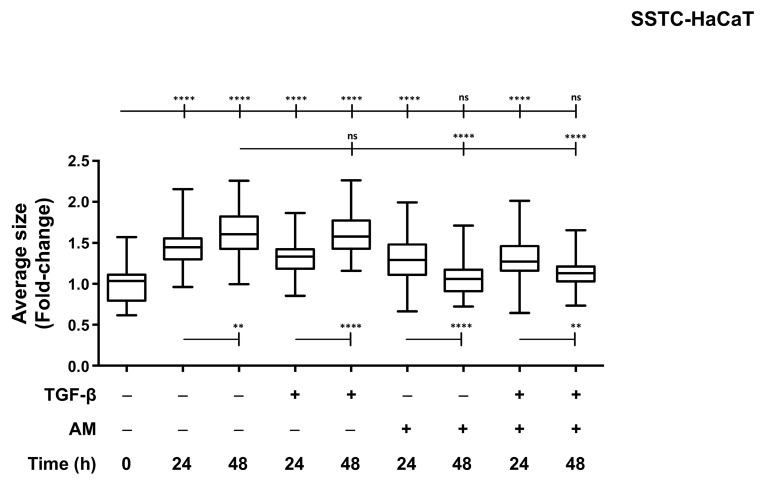
Amniotic membrane treatment restricts swelling responses in HaCaT cells persistently exposed to TGF-β. The morphology and size of monolayer cells at the wound edge were studied. Results are shown as fold-change of SSTC-HaCaT cells regarding the same parameters of SS-HaCaT cells and in relation to adjuvant treatments. Replicates from three independent experiments were analyzed. Shown data represent the mean ± SEM. Asterisks denote statistically significant differences (** *p* < 0.005; **** *p* < 0.00005); ns, non-significant.

During stimulation with TGF-β to produce SSTC-HaCaT cells, we observed that cells grew in size [18]. Additionally, as shown in Figure 1, SSTC-HaCaT cells at the wound edge looked bigger than their SS-HaCaT equivalents at the time of doing the wounding and continued to enlarge at the wound edge during the 24-h wounding assay. In order to isolate this phenomenon from the results obtained for migration during AM treatment, we measured the average cell size of SSTC-HaCaT cultures under TGF-β and/or AM treatment at both 24 h and 48 h (Figure 4). Interestingly, we found that the enlarging process in response to TGF-β continued in samples pretreated with TGF-β and later deprived of TGF-β and in those treated with TGF-β for 24 and 48 h. Strikingly, we found that in the presence of AM, with or without TGF-β, cell size recovered after 48 h of AM treatment (Figure 4). This suggests that gains in wound recovery, measured as cell migration (see Figure 3A), showed by SSTC-HaCaT treated with AM might be independent of keratinocyte size changes. Moreover, the reduced size detected at 48 h suggests that AM treatment may be capable of reverting some of the consequences of the TGF-β chronification process.

**Figure 5:**
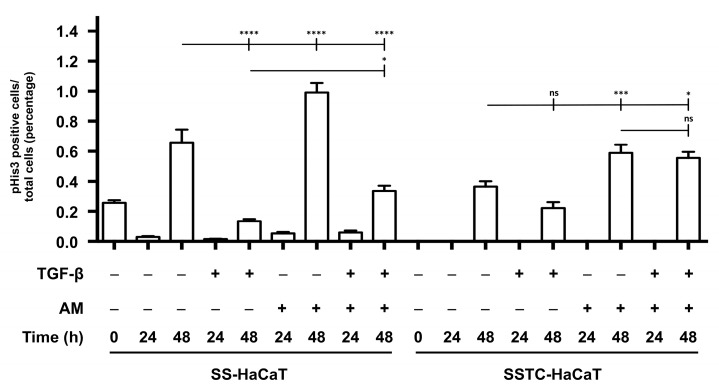
Amniotic membrane promotes cell division at the wound edge of HaCaT-cells persistently exposed to TGF-β. Quantification of the proliferation of wounded in vitro monolayers evidenced through immune labeling. Reference pictures were taken for the indicated times for each condition and treatment. Replicates from three independent experiments were analyzed. Shown data represent the mean ± SEM. Asterisks denote statistically significant differences (* *p* < 0.05, *** *p* < 0.0005, **** *p* < 0.00005); ns, non-significant.

To better characterize the recovery of wound repair capabilities after AM treatment, we tried to account for its potential contribution to cell proliferation. In our experience [18], HaCaT cells cultured either under serum starvation conditions or in the presence of TGF-β develop a state characterized by cell cycle arrest and thus a complete halt on proliferation, a similar state to that achieved upon the confluence of HaCaT cells because their growth is cell-to-cell contact inhibited (Supplementary Figure S1). For this purpose, we studied the presence of phosphorylated-Histone 3, as a proliferation marker, in the wound edge of both SSTC- and SS-HaCaT cultures under TGF-β and/or AM treatment for a period of 24 h and 48 h. A clear difference between both cultures was found right after wounding because the SS-HaCaT cultures showed some proliferative activity at the reference initial time, while SSTC-HaCaT did not (Figure 5). Interestingly, in all instances, SS-HaCaT cells showed marginal proliferation at 24 h while this activity surged at 48 h. Although conditions, including TGF-β, did follow the mentioned pattern, instead, they showed poorer values (Figure 5). Similarly, we could not track proliferation at 24 h in SSTC-HaCaT, but proliferation surged at 48 h for all study conditions, although showing lower values compared to SS-HaCaT cells (Figure 5). For the case of AM and TGF-β treatments, while the latter alone showed a non-significant detrimental effect at 48 h, AM, either alone or combined with TGF-β, showed reawakened proliferation in SSTC-HaCaT at similar levels to those of non-treated SS-HaCaT cells (Figure 5). These data indicate that SSTC-HaCaT epitheliums show a decreased capability to mount effective proliferative reactions in response to wounding challenges. While treatment with AM on wounded SSTC-HaCaT seemed to allow for improved proliferation responses, these seemed to occur late during the wound recovery process.

### 2.5. Amniotic Membrane Treatment Effectively Recovers the Proliferative Potential of Sub-Confluent TGF-β Chronified HaCaT Cells

**Figure 6:**
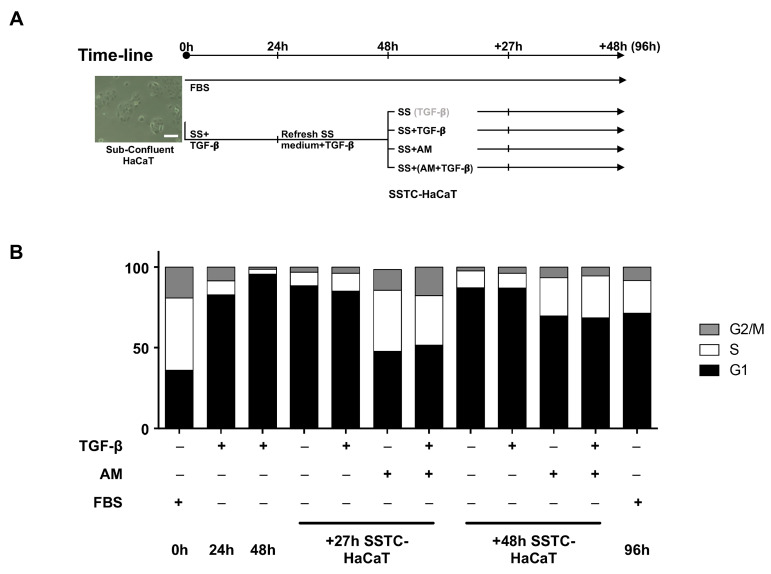
Amniotic membrane treatment reignites the proliferation of keratinocytes despite strong cell-cycle arrest status. (**A**) Illustrative scheme of the subconfluent HaCaT cells culture setup used. (**B**) Cell cycle progression was assessed through flow cytometry. Cells were analyzed at the indicated times for each condition and treatment. Replicates from three independent experiments were analyzed and represented. The scale bar equals 100 µm.

As pointed out earlier, the cell cycle arrest that characterizes SSTC-HaCaT cells reflects that of regular confluent HaCaT cultures. In order to establish whether the high confluence status of SSTC-HaCaT cultures could be affecting some of the responses registered for treatments, we developed a long-term series of assays to accurately determine proliferative responses in sub-confluent SSTC-HaCaT island cultures subject to both AM and/or TGF-β treatments (Figure 6A). On this occasion, 27 h was selected as the intermediate time point based on an incidental observation, suggesting that this little time frame difference would be more favourable to register proliferation. Interestingly, untreated SSTC-HaCaT cells showed a slight proliferative activity in this setting, with the same intensity at 27 h and 48 h. Similar results were obtained when cells were treated with TGF-β alone (Figure 6B). However, in the case of treatments, including AM, alone or combined with TGF-β, we found a robust cell cycle activation that showed a cell fraction distribution at 27 h similar to that of regular HaCaT cells and which remained robust at 48 h showing a cell fraction distribution similar to that of regular HaCaT cells as they approached confluence at 96 h (Figure 6A). These data suggest that AM can overcome the cell cycle arrest conditions imposed by TGF-β, even in the chronic environment created by continuous stimulation with TGF-β. Moreover, these data and the observations from confluent culture SSTC-HaCaT cells suggest that the potency and extent of SSTC-HaCaT keratinocyte responses to AM treatment might be greatly influenced by the epithelial conformation.

### 2.6. Amniotic Membrane Treatment Promotes Short- and Long-Term Changes in TGF-β Chronified HaCaT Cell Gene Expression

**Figure 7:**
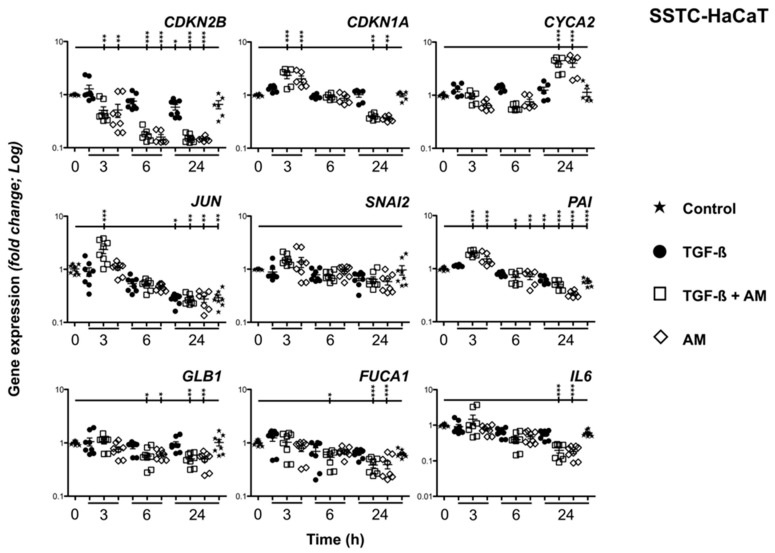
Amniotic membrane treatment ameliorates the gene expression pattern of SSTC-HaCaT cells (persistently exposed to TGF-β). Genes related to cell cycle (*CDKN2B*, *CDKN1A*, *CYCA2*), migratory responses (*JUN*, *SNAI2*, *PAI*), and senescent status (*GLB1*, *FUCA1*, and *IL-6*) were studied. Samples were obtained at the indicated times for each condition and treatment. Expression level data are represented as fold change from the initial time. Replicates from three independent experiments were quantified by qPCR. Shown data represent mean ± SEM. Asterisks denote statistically significant differences (* *p* < 0.05, ** *p* < 0.005, and *** *p* < 0.001).

In different previous works, we demonstrated the ability of AM treatments to promote distinct changes in regular HaCaT cell gene expression [22,23]. In the case of SSTC-HaCaT gene expression, these cells are characterized by an expression pattern concordant with an arrested cell cycle state or cytostasis, the acquisition of senescence and chronic inflammation features, and the development of partial epithelium-to-mesenchymal transition (EMT) [18]. This time, treating SSTC-HaCaT cells with fresh TGF-β alone did not promote remarkable changes at any study time (Figure 7). On the contrary, treatments including AM, either alone or combined with TGF-β, promoted changes that clustered expression responses of the studied genes depending on their functional involvement. First, genes participating in migration and EMT responses to TGF-β, *JUN*, *SNAI2*, and *PAI* show a shared pattern characterized by early induction after 3 h, which progressively declines at 6 h and 24 h (Figure 7). In the case of genes involved in cell regulation growth arrest *CDKN1A* (p21) and *CDKN2B* (p15), both exhibited a sharp decrease over time upon treatment with AM. On the contrary, *CYCA2*, which coordinates cell cycle progression, shows a patent overexpression at 24 h but not earlier (Figure 7). Finally, genes regarded as senescence and/or inflammation markers, *GLB1*, *FUCA1*, and *IL6*, displayed a common pattern upon AM treatment characterized by a significant reduction in gene expression levels at longer study times (Figure 7). These results reinforce the notion that AM treatments promote not only short-term responses mechanistically but also induce longer-term changes in gene expression, which can shape upcoming cell behavior. In this sense, the precise expression responses shown here might suggest some form of remission of the chronified status of SSTC-HaCaT cells upon stimulation with AM.

### 2.7. Keratinocyte Activation Markers Evolve Similarly Both In Vitro and In Vivo in Response to AM Treatment

In our experience, most molecular markers that can be easily tracked in in vitro models provide poor information when studied on biopsy samples due to differences in the tissue structure and unavoidable discrepancies during the fixation process. In order to contrast the accumulated knowledge on the SSTC-HaCaT responses to AM treatment in an in vivo human model, we studied the presence of Keratin 17 (K17), which is regarded as a marker of epithelial activation [34,35,36,37], on wounded SS- and SSTC-HaCaT cell cultures and biopsies obtained from patients undergoing compassionate treatment of diabetic foot ulcers with AM.

**Figure 8:**
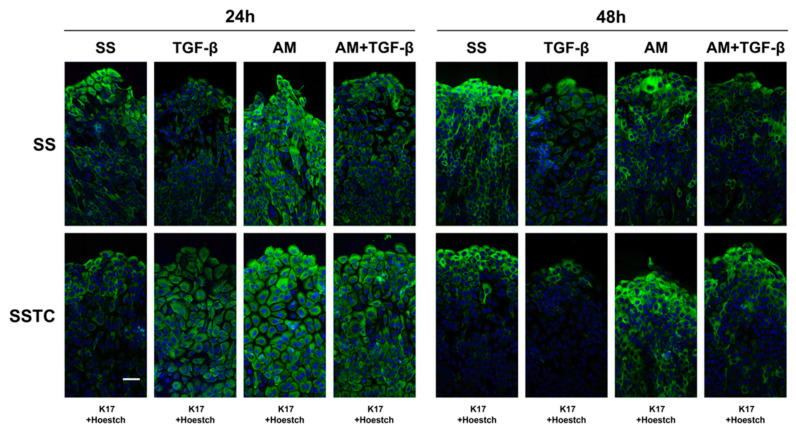
Amniotic membrane treatment reinstates functional activation of HaCaT cells persistently exposed to TGF-β. The ability of keratinocytes to develop proper activation responses upon monolayer disruption was evidenced via keratin-17 (green) immune labeling. Nuclei are colored blue. Representative images taken at the indicated times for each condition and treatment of at least three independent experiments are shown. The scale bar equals 50 µm.

In the case of wounded cultures, SS-HaCaT cells showed a similar pattern for K17 presence whether they were studied 24 h or 48 h after treatment. Untreated cells showed increased K17 presence at the wound edge in comparison with farther areas and slightly higher levels at a later time. Interestingly, the treatment with AM extended K17 increased detection to a wider area adjacent to the wound edge, especially at 24 h. On the contrary, TGF-β treatment, alone or combined with AM, greatly impaired K17 accumulation at any time (Figure 8). When observing SSTC-HaCaT cells, we found dissimilar patterns between 24 h and 48 h studies. For untreated SSTC-HaCaT, cells at the wound edge showed scarce K17 presence compared to wounded SS-HaCaT cultures, both at 24 h and 48 h, although in this case, K17 detection was higher at 48 h. In the case of AM treatments, alone or combined with TGF-β, SSTC HaCaT, and SS HaCaT developed extended K17 presence, perhaps preserving higher levels than SS HaCaT cells at 48 h. Notwithstanding, SSTC HaCaT cells treated with TGF-β also showed extended K17 presence at 24 h, strikingly less intense at the wound edge, but again, at 48 h, this treatment resulted in limited K17 detection in comparison to the rest of the conditions (Figure 8). These results fully describe how K17 presence evolves in chronified (SSTC-HaCaT) cells in response to wounding and in the presence of referenced treatments.

**Figure 9:**
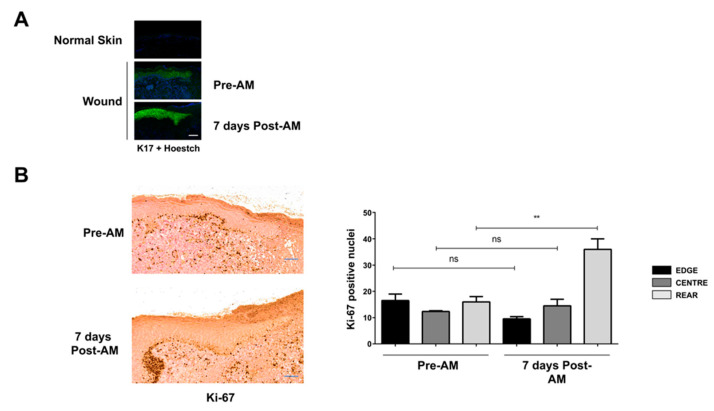
Amniotic membrane effects on chronic wounds correlate well with the observation obtained in vitro on HaCaT persistently exposed to TGF-β. Immune-histological study of human samples obtained from the edge of chronic wounds before and after amniotic membrane treatment. (**A**) Analysis of the keratinocyte activation using keratin-17 labeling (green). Nuclei are colored blue. (**B**) Analysis of the keratinocyte proliferation using Ki-67 labeling (brown deposits). Replicates from three independent samples were analyzed. Shown data represent mean ± SEM. Asterisks denote statistically significant differences (** *p* < 0.005). The scale bar equals 200 µm; ns, non-significant.

Using human biopsies, we found that normal skin is almost devoid of K17, which could be expected for an activation-specific marker (Figure 9A). In the case of biopsies taken from the wound edge of untreated chronic wounds, showing the granulation tissue area, we found a clear but diffuse presence of K17 through all the living strata of the epithelial layer (Figure 9A). Fortunately, the study of biopsies taken from chronic wounds treated with AM showed a patent K17 increase at all levels, along with a progressive increase in thickness of the epithelial layer and a clear featured keratinocyte tongue (Figure 9A). These observations do suggest that keratinocyte responses to AM treatment in chronic wounds are in line with those we observed in chronified cultured HaCaT cells.

To further support the relation suggested above, we studied proliferative responses in human biopsies using the Ki-67 marker, a nuclear protein whose presence increases with cellular proliferation. In the case of untreated biopsies, we found that Ki-67 levels were similar throughout the epithelial layer extension (Figure 9B). Interestingly, biopsies taken 7 days after AM treatment showed a patent increase of Ki-67, although restricted to areas far from the wound edge. These data would add to the notion that keratinocyte responses to AM treatment in chronic wounds align with those we observed in chronified cultured HaCaT cells, indicating that changes in cell proliferation develop with some delay.

## 3. Discussion

In the recent past, our lab showed how HaCaT cells exposed to TGF-β for a long term in the absence of serum (SSTC-HaCaT) developed features compatible with those shown by keratinocytes in chronic wounds, while HaCaT cells deprived of serum (SS-HaCaT) for the same time showed no relevant changes regarding their morphology and cellular markers [18]. However, no functional characterization was performed at that time. In this paper, we have shown how the chronification process resulting from prolonged exposure to TGF-β does hamper the functional capabilities of keratinocytes that are crucial for wound healing. Moreover, in line with our group track record, we can now confirm the ability of AM treatment within the SSTC-HaCaT setting to promote proper keratinocyte function, providing additional research evidence that supports observed results in human chronic wounds treated with AM at the clinic.

When we challenged SSTC-HaCaT epithelial continuity by artificial in vitro wounding, these cells effectively migrated less compared with control SS-HaCaT. This observation somewhat surprised us because SSTC-HaCaT develops bigger and more numerous cell protrusions [18], perhaps hinting at migratory readiness. However, as we already know, SSTC-HaCaT cells develop a strengthened cytoskeleton and increased e-cadherin tight junctions [18], which can help to explain such sub-optimal performance. However, also related to migration, when checking at the molecular level, we observed that SSTC-HaCaT cells experienced impaired FA dynamics involving paxillin and FAK. Since FA dynamics are fundamental for successful migration [29], the initial build-up of paxillin could be expected to translate into a delayed migratory onset in this setting. However, we believe improper FAK activation and subsequent dynamics arising from the chronified status might be the main factor driving SSTC-HaCaT cell sub-optimal migratory performance. It is worth highlighting that since these cells, both SSTC-HaCaT and SS-HaCaT, experience a strong cell cycle arrest [18], the wound recovery level achieved should be attributed just to migration. However, crippled dynamics observed in the chronified state would be a sign of failure to properly activate upon injury and in the long term. Since keratinocyte migration constitutes the key step for successful epithelial closure and our observations clarify SSTC-HaCaT impaired behavior in this regard, these results might further strengthen the validity of our proposed serum-starved long-term TGF-β-exposed HaCaT chronified cell-model for the in vitro assessment of impaired wound healing.

As mentioned in the introduction, our lab accumulates a solid research track record about the use of amniotic membrane (AM) as a treatment for epithelial wounds [19,20,21,22,23,24]. Although patients have an almost 100% recovery rate for treated complicated wounds, evidence is very limited due to the compassionate and exceptional nature of the procedure [24]. Nevertheless, by resorting to conventional in vitro models, we were able to demonstrate the ability of AM treatment to rapidly trigger FA remodeling and promote cell migration [21]. However, conclusions in that conventional setting should be considered carefully since the culture medium comprised fetal bovine serum (FBS) at that time, which is known to contribute to the migration and proliferation of HaCaT cells. In this regard, this study did not use FBS during the conditioning phase. It was only used later as a control treatment because FBS had the potential to provide more specific results for each treatment. As such, we found that AM restated the wound recovery performance of SSTC-HaCaT cells to levels comparable to SS-HaCaT control cells under similar conditions. Regarding FBS, we previously knew that its addition to SSTC-HaCaT promotes an increase in cell size and the development of potentiated EMT features [18], but we were unaware of any effects on migration or proliferation. Surprisingly, in this study, while treatment with FBS proved ineffective for SS-HaCaT controls, it was clearly detrimental to migration for SSTC-HaCaT cells. As FBS-exposed SSTC-HaCaT cells developed features compatible with EMT, perhaps enduring such phenotype morphing could also hamper migration. Interestingly, we observed how SSTC-HaCaT cells at the leading edge experienced a swelling-like increase in size upon wounding, although there were no potentiated EMT signs for regular SSTC-HaCaT or for AM-treated cells. The concept of EMT implies a fundamental phenotypical change that provides epithelial cells with unusual capacities, including resistance to apoptosis, enhanced mobility, and the ability to surpass basement membranes. It is worth noting that while potentiated EMT states are considered an unequivocal trait of advanced tumor transformation [38], there is controversy on the need for partial and intermediate EMT states to develop regeneration processes and wound healing [39]. In this regard, our results would provide further evidence that AM treatment at the clinic is safe. Our previous works indicated that TGF-β signaling was required to obtain strengthened responses to AM [23]. However, within the current setting, TGF-β presence during AM exposure did not seem to influence SSTC-HaCaT migration, while it had a slightly detrimental effect on control SS-HaCaT cells. In this sense, although remnants of TGF-β may persist in every chronified setup culture medium, these results generate additional evidence of the AM capability to fine-tune responses to TGF-β even in such saturated environments. At the molecular level, it was interesting how FAK activation and FA dynamics in AM-treated SSTC-HaCaT cells resembled those found in control cells. Jointly, these observations suggest that, within our in vitro chronified keratinocyte setup, AM treatment does in fact promote powerful responses, thus being capable of reverting some of the consequences of chronification without the risks associated with potentiating EMT.

Next, we tried to ascertain whether AM contribution to migration was due to changes in morphology and/or to a restoration of the characteristic proliferation disturbances of SSTC-HaCaT cells. Within the same setting used to study migration, both for SS-HaCaT and SSTC-HaCaT, cell cycle arrest was achieved due to serum starvation, contact inhibition, and/or cytostasis promoted by TGF-β. Using cytochemical labeling, we found that injuring the monolayer in SS-HaCaT cells did promote the activation of proliferation, despite the absence of serum and similarly to regular scratched HaCaT monolayers [20,21,22,23]. On the contrary, on SSTC-HaCaT cells, there was no cytochemical labeling and thus no proliferation, even after the wounding, probably because of persistent cytostasis induced by TGF-β. For the case of AM-exposed samples, the treatment allowed SSTC-HaCaT cells to escape cycle arrest even in the presence of fresh TGF-β. However, this occurred only after long treatment periods of AM exposure (48 h), which probably means these cells need time to undergo machinery readjustments. Interestingly, developing a setup expanded in time allowed us to better characterize the swelling-like response detected in cells at the leading edge of the wound, and we found that AM treatment prevented SSTC-HaCaT cells from fully swelling (24 h) and it even slightly reduced their size after long periods (48 h). However, to see if AM was capable of reverting the consequences of chronification over proliferation, we decided we should study that parameter again, this time on subconfluent SSTC-HaCaT cells to properly separate the effects derived from TGF-β cytostasis from those related to contact inhibition. Notably, within the subconfluent setup, just slight changes in proliferation were found both in untreated cells and in cells exposed to fresh TGF-β. These results are in line with previous observations [18]. Strikingly, within the subconfluent setup, AM effects on proliferation seemed to start to surface 27 h after exposure, quite earlier than in the wound recovery assay. This again suggests that some time might be necessary for SSTC-HaCaT cells to undergo machinery readjustments. Nevertheless, SSTC-HaCaT cells seemed to experience a status prone to swiftly scape cytostasis upon proper stimulus [18], although these cells should still show altered cytoskeletal and molecular features. All these observations again add to the notion that AM does restore the functional capabilities of chronified SSTC-HaCaT keratinocytes and also indicate that early gains in migration might be independent of changes in morphology or proliferation reactivation but might also benefit from them later.

As already mentioned, SSTC-HaCaT cells developed an altered gene expression pattern [18]. As such, if AM treatment did alleviate some of the consequences of TGF-β mediated chronification, this effect could be expected to also involve the modulation of gene expression. More precisely, we studied the expressions of known sets of genes involved in cell cycle progression, migratory activity, and inflammation/senescence. Interestingly, while *JUN*, *SNAI2*, and *PAI* showed an expression pattern characterized by early induction just 3 h after AM treatment, *CDKN2B* (p15), *CDKN1A* (p21), and *CYCA2* showed longer response dynamics, especially the cyclin gene, where the levels are fundamental for a successful G2/M transition [40]. These observations are indeed in line with functional observations of the effects of AM treatment, manifested as an early activation of migration and a later resumption of cell cycle and proliferation requiring some machinery readjustment to proceed. All three genes studied related to senescence and inflammation, *GLB1*, *FUCA1*, and *IL-6*, developed longer-term evolving patterns characterized by significantly reduced levels. It is worth noting that IL-6 is considered to play a fundamental role in sustaining senescence as part of its associated secretome [41,42]. In this sense, high IL-6 levels have been found to correlate with delayed wound healing in tuberculosis patients [43]. Moreover, the AP-1 family of transcription factors, including c-Jun, has been suggested to regulate the expression of the *IL-6* gene. We want to mention that we showed the consequential activation of c-Jun resulting from persisting TGF-β signaling [18] in our previous study in this research line. We also showed that SSTC-HaCaT cells develop an increased expression for all three TGF-β isoform genes and also *IL-6* [18]. Thus, our data then indicated the existence of intricate crosstalk involving IL-6 and TGF-β autocrine loops contributing to the build-up of senescent responses typical of keratinocytes in chronic wounds. To this extent, our current results suggest that, upon AM treatment, SSTC-HaCaT keratinocytes might be released from the local conditioning caused by both autocrine pro-senescence and pro-inflammatory loops, probably thanks to a fine-tuning of the TGF-ß signaling as described in non-chronified HaCaT cells in the past [23]. This, of course, adds to the notion that AM treatment could be able to reset, at least partially, the chronified state of SSTC-HaCaT cells.

One striking feature of chronified SSTC-HaCaT cells that we found is that, despite being keratinocytes, they show poor keratin contents [18]. In humans, up to 54 functional keratin genes have been described. These are expressed in very precise spatial and temporal patterns related to the epithelial nature of cells and their stage of differentiation, constituting an integral building block of the epithelial cytoskeleton, which is crucial for the integrity and mechanical stability of epithelial cells and tissues. Furthermore, some keratins have been found to show regulatory functions, participating in different intracellular signaling pathways, including those related to stress and wound healing [36]. Interestingly, others have also described how persistent TGF-β signaling is related to altered expression of different keratin genes and proteins, more precisely, the keratins of stratified squamous epithelia keratin-5 and keratin-14 [44,45]. These two keratins constitute the primary blocks providing physical stability to epidermal keratinocytes [36], so an overall poor keratin content in SSTC-HaCaT cells could consequently be the sign of a decreased structural stability related to a senescent/swollen state. In this sense, the expression of keratin-17 is known to be a specific marker of epidermal keratinocyte activation related to wound recovery, as its expression is known to be triggered upon wounding [34,35,36,37]. In this report, we show how untreated chronified SSTC-HaCaT cells experienced poor activation upon wounding. Notably, the functional importance of such a finding has been hinted at by another study in which keratin-17 knockout mouse embryos experienced a delay in the closure of surface ectoderm wounds [34]. Similar to other analyses in this study, in vitro AM treatment of SSTC-HaCaT cells seemed to restore their capacity to activate upon wounding, as revealed by keratin-17 induction. This finding was of utmost importance because it had the potential to link the observations made both in vitro and in the clinic. This is driven by the limited availability of samples of human chronic wounds treated with AM due to the compassionate nature of this procedure. Fortunately, we were able to confirm that keratinocytes located at the edges of chronic wounds did show similar keratin-17 labeling as in our SSTC-HaCaT experimental setting, revealing a sharp increase in its expression resulting from AM treatment. Moreover, these samples also revealed a recovery in proliferation based on Ki-67 immuno-labeling reading. These results suggest that the mechanisms involved in our chronified SSTC-HaCaT cell model and in the pathological physiology of keratinocytes in human chronic wounds would be similar, always bearing in mind that sustained TGF-β levels might be a common factor.

If we take into account all of the above, the results of this work present a quite complete picture of how sustained TGF-β might play a major role in wound chronification and provide strong evidence of the validity of our TGF-β chronified HaCaT in vitro model (SSTC-HaCaT) for the study of chronic wound physiology. Moreover, based on this model, here we provide data and observations that validate the behavior of the SSTC-HaCaT model in relation to the treatment with AM and previous observations obtained regarding such treatment for regular HaCaT cells. In this sense, as a late note regarding the powerful observations made here, we want to emphasize that the effects of AM on SSTC-HaCaT should be considered somewhat partial or perhaps biased if assessed in comparison to an in vivo model. In such cases, the epithelial compartment and underlying tissue layers would respond jointly to the treatment, which would be critical in the case of blood vessels in the dermis. This is because they constitute the main source of nutrients and a key hub for necessary feedback to be traded with fibroblast and immune cells, thus contributing to the whole healing process. In this line, our group is researching the effects of AM treatment on endothelial cell physiology with promising results [46]. Nevertheless, we are certain about the validity of the proposed model as a proper complicated wound in vitro epithelial surrogate. Thus, we encourage the scientific community to embrace it for the advancement of the research on chronic wounds’ physiology and healing.

## 4. Materials and Methods

This study has been approved by the ethics committees at the University Clinical Hospital Virgen de la Arrixaca (Murcia, Spain) and the Spanish Agency for Drugs and Medical Devices (AEMPS). In every case, appropriate written informed consent was obtained from the AM donors.

### 4.1. AM Processing and Treatments

Amniotic membrane was prepared as described in [22]. Briefly, full-term placenta from healthy donor mothers was obtained from uncomplicated cesarean section. The fetal membranes were washed in Physiological Saline Solution (PSS) (B. Braun, Barcelona, Spain) supplemented with 50 µg/mL Amphotericin (Bristol-MyersSquibb, Madrid, Spain), 48 µg/mL Clotrymazol (Almirall-Prodesfarma, Barcelona, Spain), 50 µg/mL Tobramycin (Laboratorios Normon, Madrid, Spain) and 50 µg/mL Vancomycin (Laboratorios Hospira, Madrid, Spain) and rapidly transferred to the laboratory in sterile conditions. Under a laminar flow cabinet, the amnion was mechanically peeled from the chorion, washed three to four times with 200 mL of PBS (Biowest, Nuaillé, France), and flattened onto sterile nitrocellulose paper (Pierce, Thermo Fisher Scientific, Waltham, MA, USA) with the basement membrane surface up, and the spongy layer facing and sticking to the nitrocellulose paper. Then, paper with adhered membrane was cut into 1 cm × 1 cm fragments. Freshly cut AM fragments were separated from paper pieces and placed in a sterile vial containing a freezing solution made of 10% Dimethyl Sulfoxide (DMSO) (Sigma-Aldrich, St. Louis, MO, USA), 4% human albumin (Grifols, Bercelona, Spain) in DMEM (Biowest, Nuaillé, France) medium and then frozen at −80 °C; later, it was preserved in liquid nitrogen until further use. On the day of application, AM pieces were thawed at 37 °C, and then they were washed three times with DMEM and placed at 37 °C in a 7.5% CO_2_ incubator for two hours for the revitalization of the AM cells. Then, the fragments were used for the desired experiment.

### 4.2. Cell Culture Conditions and Chronification

HaCaT cells [47], obtained from ATCC, were cultured in full medium (FM) comprising Dulbecco’s modified eagle high-glucose medium (Biowest, Nuaillé, France), completed with 10% FBS (Thermo Fisher Scientific, Waltham, MA, USA), 1000 units/mL penicillin, 1000 μg/mL streptomycin (Sigma-Aldrich, St. Louis, MO, USA), and 1% l-glutamine (Biowest, Nuaillé, France), at 37 °C in a 7.5% CO_2_ controlled atmosphere. Chronification was as indicated in Liarte et al., 2020 [18]. Briefly, HaCaT cells at 50% subconfluency were changed to a serum-deprived medium (SS). While some of them were kept in this condition, others were stimulated with 2 ng/mL of TGF-β1 (PeproTech, Rocky Hill, NJ, USA). For samples maintained continuously in the absence or presence of TGF-β, a culture medium supplemented with 2 ng/mL of TGF-β1 was refreshed every 24 h. After 48 h of treatment, cells were denominated SS (serum-starved) or SSTC (serum-starved TGF-β chronic) cells [18], respectively. Then, cells were treated for the conditions stated in each experiment.

### 4.3. Islet Cell Density and Cell Size 

Reference images from HaCaT cells subjected to different treatments were obtained using Moticam camera 2300 3.0 M Pixel USB 2.0 coupled with an optical microscope (Motic Optic AE31 from Motic Spain, Barcelona, Spain), and applying matching picture settings for magnification and digital resolution. To determine the islet cellular density values, a total of fifty cell islets per condition were characterized using ImageJ Fiji software (Version: 2.9.0/1.53t) distribution and applying area drawing and cell counting functions [48].

### 4.4. Wound Healing Scratch Assay 

HaCaT cells were seeded in 24-well plates until they reached 100% confluence. Cells were then subjected to the chronification method for 48 h until they reached the status of SS or SSTC, SS-HaCaT, and SSTC-HaCaT, respectively. At the initial time (T0), a cross-shaped scratch was made on the cell monolayer using a sterile 200 µL micropipette tip. The original medium where cells had been grown was kept, and extensive washing with PBS and FBS-free culture medium was conducted to wash out released or unattached cells. The original culture medium was centrifuged to clear out cell debris and floating cells and was replaced back into either SS or SSTC cells. Then, one 1 cm^2^ AM piece was added per well on top of the migrating cells and corresponding treatments with either TGF-β or a combination of both AM and TGF-β. Cell migration was measured by taking pictures at the beginning, before the addition of AM, and at the end of the experiment at the hours indicated in each experiment using 10× magnification with a Moticam camera 2300 3.0 M Pixel USB 2.0 coupled with an optical microscope (Motic Optic AE31 from Motic Spain, Barcelona, Spain). Images were processed using ImageJ Fiji software (Version: 2.9.0/1.53t) software to draw the cell-free region limits in each case. The initial cell-free surface was subtracted from the endpoint cell-free surface, and the resulting values were plotted in a graph.

### 4.5. Immunocytochemistry and Image Analysis

HaCaT cells were grown to 100% confluence over round glass coverslips in several 10-cm diameter plates. Cells were then subjected to the chronification method for 48 h until they reached the SS or SSTC status, SS-HaCaT, and SSTC-HaCaT, respectively. Before the wounding, the original medium where cells had been grown for 48 h was removed, kept, and centrifuged to clear out cell debris and floating cells and reserved for further use. Just before the assay, coverslips with cells were placed in an FBS-free medium where epithelial monolayers on coverslips were wounded with a razor blade, which was immediately dragged to create a space large enough to allow migration. Then, coverslips with attached cells were placed into a six-well plate FBS-free medium. When all coverslips had been processed, the medium was again replaced with reserved medium from 48 h culturing conditions of either SS- or SSTC-HaCaT cells. At T0, pieces of AM (six of 1 cm^2^) were added per plate over the culturing medium of the wounded cells, or 2 ng/mL of TGF-β or a combination of both AM and TGF-β. After the indicated times on each experiment, the medium was removed, and coverslips were fixed with 4% formaldehyde (Applichem GmbH, Darmstadt, Germany) in PBS. Subsequently, cells were permeabilized using 0.3% Triton X-100 (Sigma-Aldrich, St. Louis, MO, USA) in PBS, and unspecific staining blocking was applied [0.3% Bovine Serum Albumin (BSA) (Santa Cruz Biotechnology, Heidelberg, Germany), 10% FBS (Thermo Fisher Scientific, Waltham, MA, USA), 0.1% Triton X-100 (Sigma-Aldrich, St. Louis, MO, USA) in PBS] supplemented with 5% skimmed milk (Beckton Dickinson, Franklin Lakes, NJ, USA). Immunostaining was performed using a blocking solution as a vehicle. The following commercial antibodies were used: anti-phospho-FAK (Abcam, Cambridge, MA, USA); anti-paxillin, anti-cytokeratin 17, and phospho-Histone3 (Santa Cruz Biotechnology, Heidelberg, Germany); anti-Ki67 (Dako Denmark, Glostrup, Denmark). For secondary immunofluorescence staining, the following antibodies were used: Alexa Fluor 488 conjugated anti-rabbit (from donkey), Alexa Fluor 488 conjugated anti-mouse (from donkey), and Alexa Fluor 594 conjugated anti-mouse (from donkey) (all from Thermo Fisher Scientific, Rockford, IL, USA). When indicated, appropriate fluorescent-conjugated secondary antibodies were used together with Alexa fluor 594-conjugated phalloidin (Molecular Probes, Thermo Fisher Scientific), where indicated, and Hoechst 33258 (Fluka, Biochemika, Sigma-Aldrich, St. Louis, MO, USA) to reveal actin, and nuclei, respectively. Images were acquired with a confocal microscope (LSM 510 META from Zeiss, Jena, Germany), and a representative image was used for illustrative purposes in the figures.

### 4.6. Quantitative PCR

Total RNA was extracted from sub-confluent (65%) HaCaT cell cultures using the RNeasy-mini system (Qiagen, Venlo, The Netherlands). Typically, 900 to 800 ng of RNA from independent samples were retro-transcribed using iScript reagents (Bio-Rad, Hercules, CA, USA). The resulting cDNA was used for quantitative PCR (qPCR) implementing the SYBR premix ex Taq kit (Takara Bio Europe/Clontech, Saint-Germain-en-Laye, France) according to the manufacturer’s instructions. For each mRNA, gene expression levels were normalized to the glyceraldehyde 3-phosphate dehydrogenase (GAPDH) content of each sample by applying the comparative Cq method (2^−∆∆Cq^). The primers used are detailed in Table S1. Replicates from three independent experiments were quantified. Analyzed data represent the mean ± SEM.

### 4.7. Cell Cycle Quantification

The cells were seeded in FM on 6 cm culture plates and grown to 35% confluence, then specific conditions for chronification were applied until SS and SSTC conditions were achieved (see above). After pre-conditioning, post-treatments were performed by introducing AM (4 pieces per well of a 6 well plate), 2 ng/mL of TGF-β, or a combination of both. FBS 10% or EGF 10 ng/mL were used alone or in combination with fresh TGF-β1 2 ng/mL inoculation for 24 h. Cells were harvested by detachment with trypsin/EDTA (Biowest, Nuaillé, France). For cell cycle analysis, samples from detached cells’ samples were centrifuged, and the resulting pellet was immediately fixed with ice-cold 70% ethanol and stored at 4 °C for future analysis. The cells were then washed three consecutive times with cold PBS (Thermo Fisher Scientific, Waltham, MA, USA) in order to remove ethanol, and they were finally stained for cell cycle assessment. Briefly, the cells were treated with a solution of 20 μg/mL Ribonuclease A and 40 μg/mL propidium iodide (all from Sigma-Aldrich, St. Louis, MO, USA) in PBS. Cells were analyzed through flow cytometry using a FACS Calibur 1 (Beckton Dickinson, Franklin Lakes, NJ, USA).

### 4.8. Statistics

Data from colony density and qPCR were analyzed with analysis of variance (ANOVA) applying Bonferroni’s multiple comparison correction and using Prism’s Graph Pad Data Analysis software (Version: 7.0a). *p*-values lower than 0.05 were considered to be statistically significant. In figure legends, asterisks denote statistically significant differences between conditions and treatments (* *p* < 0.05, ** *p* < 0.005, *** *p* < 0.001, and **** *p* < 0.0001).

## Supplementary Materials

The following supporting information can be downloaded at: https://www.mdpi.com/article/10.3390/ijms24076210/s1.
