# Supplementary material for: Amniotic Membrane Restores Chronic Wound Features to Normal in a Keratinocyte TGF-β-Chronified Cell Model
Source: Int J Mol Sci. 2023 Mar 25;24(7):6210. doi: 10.3390/ijms24076210 (PMC10094701; doi:10.3390/ijms24076210)
Supplement: Supplementary file 1 [file ijms-24-06210-s001.zip › ijms-2252172-supplementary.pdf]

International Journal of
Molecular Sciences

MDPI

Amniotic Membrane Restores Chronic Wound Features to Normal in a Keratinocyte TGF-β-Chronified Cell Model

Sergio Liarte [1,2,*], Ángel Bernabé-García [1], Mónica Rodríguez-Valiente [1,2], José M. Moraleda [3], Gregorio Castellanos [4] and Francisco J. Nicolás [1,*]

1 Laboratorio de Regeneración, Oncología Molecular y TGF-β, IMIB-Arrixaca, El Palmar, 30120 Murcia, Spain
2 Advanced Therapies in Regenerative Medicine Based on Molecular and Cellular Biology, Universidad Católica de Murcia (UCAM), Campus de los Jerónimos, Guadalupe, 30107 Murcia, Spain
3 Unidad de Terapia Celular, Hospital Clínico Universitario Virgen de la Arrixaca, El Palmar, 30120 Murcia, Spain
4 Unidad de Heridas Crónicas y Úlcera de Pie Diabético, Hospital Clínico Universitario Virgen de la Arrixaca, El Palmar, 30120 Murcia, Spain
* Correspondence: sdll1@um.es (S.L.); franciscoj.nicolas2@carm.es (F.J.N.)

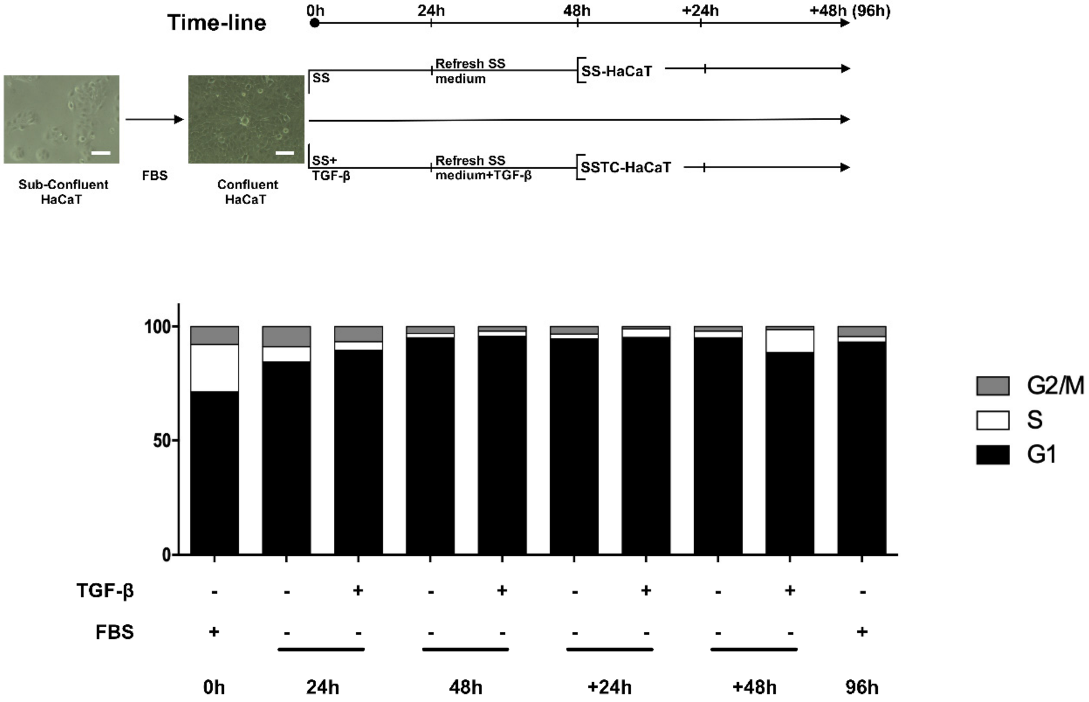

Figure S1. HaCaT confluent cells cultured under serum starvation conditions or in the presence of TGFß experience a G1 cell cycle arrest and a complete halt on proliferation. The scheme describes how HaCaT cells were cultured in serum until they reached confluency. Then, they were deprived from serum and treated or not with TGF-ß. Cell cycle progression of each stage was assessed through flow cytometry. Cells were analysed and the indicated times for each condition and treatment. Replicates from three independent experiments were analysed and represented. Bar equals 100 μm.

Table S1. Different primers used to study the expression of several genes.

**Primer Name**	Primer sequence 5'to 3'
GAPDH-Fwd	ACCACAGTCCATGCCATCAC
GAPDH-Rev	TCCACCACCCTGTTGCTGTA
PAI-Fwd	GACATCCTGGAACTGCCCTA
PAI-Rev	GGTCATGTTGCCTTTCCAGT
JUN-Fwd	GGAAACGACCTTCTATGACGATGCCC
JUN-Rev	GGCGCGCACGAAGCCCTCGGCGAACC
SNAI2-Fwd	GATGCCGCGCTCCTTCCTGGTC
SNAI2-Rev	GCTGCTTATGTTTGGCCAGCC
CDKN2B-Fwd	ATGCGCGAGGAGAACAAG
CDKN2B-Rev	CTCCCGAAACGGTTGACTC
CDKN1A-Fwd	ATGTCAGAACCGGCTGGGGATG
CDKN1A-Rev	GGGCTTCCTCTTGGAGAAGATC
GLB1	Proprietary sequence (Sigma KiCqStart)
IL6	Proprietary sequence (Qiagen QuantiTect)
CYCA2	Proprietary sequence (Sigma KiCqStart)
FUCA1-Fwd	AGTCACCCTGTTGCCTATGG
FUCA1-Rev	TTTGGCGCTTTTAGATTGCT
